# Giant NLO response and deep ultraviolet transparency of dual (alkali/alkaline earth) metals doped C_6_O_6_Li_6_ electrides

**DOI:** 10.1016/j.heliyon.2023.e18264

**Published:** 2023-07-13

**Authors:** Naveen Kosar, Sunaina Wajid, Khurshid Ayub, Mazhar Amjad Gilani, Nur Hazimah Binti Zainal Arfan, Malai Haniti Sheikh Abdul Hamid, Muhammad Imran, Nadeem S. Sheikh, Tariq Mahmood

**Affiliations:** a Department of Chemistry, University of Management and Technology (UMT), C-11, Johar Town Lahore, Pakistan; b Department of Chemistry, COMSATS University Islamabad, Abbottabad Campus, Abbottabad-22060, Pakistan; c Department of Chemistry, COMSATS University Islamabad, Lahore Campus, Lahore, Pakistan; d Chemical Sciences, Faculty of Science, Universiti Brunei Darussalam, Jalan Tungku Link, Gadong BE1410, Brunei Darussalam; e Department of Chemistry, Faculty of Science, King Khalid University, P.O. Box 9004, Abha 61413, Saudi Arabia; f Department of Chemistry, College of Science, University of Bahrain, P.O. Box 32038, Bahrain

**Keywords:** Electride, Nonlinear optics, DFT, First hyperpolarizability, Three-level model

## Abstract

The designing of new materials having outstanding nonlinear optical (NLO) response is much needed for use in latest optics. Herein, the geometric, electronic and NLO properties of alkali and alkaline earth metals doped C_6_O_6_Li_6_ (alk-C_6_O_6_Li_6_-alkearth, alkearth = Ca, Mg, Be and alk = K, Na, Li) electrides is studied via quantum chemical approach. The interaction energies (E_int_) are examined to illustrate their thermodynamic stability. The strong interaction energy of -39.99 kcal mol^-1^ is observed for Ca–C_6_O_6_Li_6_–Li electride in comparison to others. Frontier molecular orbitals (FMOs) energy gap of considered complexes is changed due to the electronic density shifting between metals and C_6_O_6_Li_6_ surface, which notifies the semi conducting properties of these electrides. The FMOs isodensities and natural bond orbital (NBO) charge analysis are performed to justify charge transfer between dopants and complexant. UV–Visible study also confirmed the application of these electrides as deep ultra-violet laser devices. NLO response is studied through calculation of first hyperpolarizability (*β*_o_). The highest *β*_o_ value of 1.68 × 10^5^ au is calculated for Mg–C_6_O_6_Li_6_–K electride. NLO response is further rationalized by three- and two-level models approach.

## 1 Introduction

The designing and synthesis of nonlinear (NLO) materials is an important area of research for chemists based on their applications in laser technologies [1], dynamic imaging [2], computing [3], optical communication [4], & optical logic [5], optoelectronic switching [6] and superior photonics [7] *etc*. Dipolar molecules are considered as effective NLO optics, afterward, scientist observed that non-dipolar molecules known as octupolar molecules are more efficient candidates for NLO optics [8].

Introduction of excess electrons with in a complex is the latest technique for enhancing the hyperpolarizability, which ultimately increases the NLO response [[9], [10], [11], [12], [13], [14], [15], [16]]. The most important parameter for estimation of NLO response is hyperpolarizability value of respective material. The system having more hyperpolarizability value is known to has better NLO response. Mostly, transition metals [17], alkaline earth metals [18], alkali metals [19], superhalogens [20,21] and superalkalis [22,23] are used as dopant for the introduction of excess electrons,. The excess electrons containing complexes are further characterized as alkalides [24] and electrides [[25], [26], [27]]. In electrides the excess electrons are not localized on any atom but occupy free space act as anionic specie and on the other side whole complex acts as cationic specie (e^−^[metal-complex]^+^) [25]. Currently, scientist observed that alkalides and electrides are the best NLO candidates for designing novel NLO materials.

Recently, large number of studies are reported demonstrating the NLO applications of electrides. Mahmood and coworkers designed Janus type electrides by doping of superalkalis on all-cis-1,2,3,4,5,6- hexafluorocyclohexane,. Na_2_F@C_6_F_6_H_6_ complex has the highest hyperpolarizability (1.68 × 10^6^ au) [28]. Kosar et al. reported an increase in the first hyperpolarizability by doping superalkali on C_6_S_6_Li_6_ cluster. Li_3_O@C_6_S_6_Li_6_ complex showed electride character and the highest first hyperpolarizability [29]. Wang et al. computationally designed On/OFF switches having electride properties and seen very high static first hyperpolarizability (4.06 × 10^5^ au) switching from Off to on form [30]. Superalkalis doped hexamethylenetetramine, new type of electrides reported by Hou et al. have hyperpolarizability values range from 0.65 × 10^3^–4.72 × 10^5^ [31]. Lu et al. worked on the NLO properties of alkali metals doped aziridine electrides, they observed the large hyperpolarizability for these electrides (3.4 × 10^6^ au) [32]. Wu and coworkers converted aluminum and phosphide doped boron nitride sheet into electrides by chemical doping of alkali metals and concluded that high hyperpolarizability values (1807–60919 au) illustrate their enhanced NLO response [33]. Zhang et al. report large NLO response and earthide characteristics of stacked dimer and trimer of Janus type-hexafluorocyclohexane (F_6_C_6_H_6_) after doping with alkaline earth metal [34]. Zhang and coworkers executed the comparative analysis of one and two Li atoms doping with B_20_H_26_ and observed the largest hyperpolarizability value of 108846 au for two lithium doped B_20_H_26_ and observed the electride properties of respective complexes [35]. Huang and coworkers investigated the NLO properties of alkali metals doped Al_12_N_12_inorganic electrides [36]. Gu and coworkers investigated Li salt of pyridazine doped with two Na atoms and resultant hyperpolarizability was 1.412 × 10^6^ au [37].

Nowadays, doping of dual metals (bis alkaline earth metals) is considered the most rational approach for enhancement of NLO response of respective system [15,38]. In dual system, low ionization potential metals easily eject their electrons toward the available surface [[39], [40], [41], [42]]. In literature multiple metals doped nanomaterials are reported those have enhanced NLO response. For example, one such dual system of BNNT encapsulated LiCN…Li complex is designed by Zhong et al. with considerable hyperpolarizability up to 1.0 × 10^4^ au along with promising electronic stability [43].

Ayub and co-workers studied F_6_C_6_H_6_ which is doped with bis-alkaline earth metals. The higher *β*_o_ values (up to 2.91 × 10^4^ au) notified these complexes as best candidates for NLO materials [44]. A remarkably higher hyperpolarizability (1.26 × 10^5^ au) is calculated by Wang et al. for Be(NH_3_)_n_M complexes (where n = 1–3 and M = Ca, Be) in comparison to Li(NH_3_)_n_Na (n = 1–3) [45]. Roy et al. theoretically studied the NLO response of binuclear sandwich electrides M^1^_2_(η^8^-C_8_H_8_)_2_M^2^_2_ (M^2^ = Mg, Ca & M^1^ = K, Na) and observed high hyperpolarizability value in the range of 2.6 × 10^5^ to 1.4 × 10^6^ au [46]. A type of dual metals earthides Met^+^(26 Adz)Met^−^ (Met^−^ = Ca, Mg, Be & Met^+^ = Na, Li) are introduced by Ahsan et al., they noticed excellent hyperpolarizability for these complexes than alkalides [47]. Kosar et al. worked on the NLO response of first three elements of 2nd group elements metals doped cages (C_20_ and C_24_) and they observed the increase in hyperpolarizability (up to 3.62 × 10^6^ au) with doping of multiple alkaline earth metal compared to single metal doping [18,48]. Similarly, they seen the same trend for C_20_ and C_24_ cages doped with multiple as well as single first three elements of 1st groups [18,49].

These reports suggested 1st and 2nd group metals are good sources of excess electrons and can efficiently improve the first hyperpolarizability. In our opinion the search for a better dual metals doped complex having extraordinary NLO response is the need of upcoming time. It is expected that if the conductive properties of organometallics are enhanced by dual doping of low ionization potential metals, which can result in better NLO response. According to our observations there is no report on NLO response of the dual metals doping on C_6_O_6_Li_6_. In this report, we used DFT method to study the NLO properties and electride characteristics of 2nd group (alkaline earth metals) and 1st group (alkali metals) doped C_6_O_6_Li_6_ complexes.

## 2 Computational methodology

Gaussian 09 software is used for simulation of designed structures [50]. Optimization of structures is performed at ωB97XD/6-31+G(d,p) method. Frequency analysis is also executed at the same method, to predict the required structures as true minima. The charge is zero for pure C_6_O_6_Li_6_ sheet and all optimized complexes. The pure C_6_O_6_Li_6_ (singlet, triplet and quintet) sheet and all optimized complexes (doublet, quartet and sextet) are optimized at three lower spin states. It is observed that the doped structures are more stable at doublet spin states whereas pure sheet is most stable at singlet spin state. To elucidate thermal stability of desired complex, interaction energy is calculated by using the following Equation (1).

(1) E_int_ = E_complex_ – (E_pure sheet_ + E_alkali metals_ + E_alkaline earth metals_)

Here, E_pure sheet_ is the energy of pure C_6_O_6_Li_6_ sheet, E_alkali metals_ corresponds to the energy of alkali metals and E_alkaline earth metals_ corresponds to the energy of alkaline earth metals. E_complex_ is the energy of dual alkali & alkaline earth metal doped C_6_O_6_Li_6_ complex. Quantitative charge transfer between these species is estimated through NBO analysis at same level of theory. between metal atom and C_6_O_6_Li_6_ surface. Equation (2) is used for calculations of vertical ionization energy (VIE).

(2) VIE = E(X^+^) - E(X)

Where, E(X^+^) and E(X) are the parameters for energy of cation and neutral dual metals doped C_6_O_6_Li_6_, respectively. The energy difference (E_H-L_) between FMOs is calculated by using the following Equation (3).

(3) E_H–L_ = E_L_ - E_H_

The linear optical and NLO responses are estimated through polarizability (*α*_o_) and first hyperpolarizability (*β*_o_) parameters, respectively which are calculated at the same ꞷB97XD, CAM-B3LYP and LC-BLYP functionals along with Pople’s basis set (6-311++G(2d, 2p)) for appropriate estimation of optical responses. The results at CAM-B3LYP, and LC-BLYP functional and Pople’s basis set (6-311++G(2d, 2p)) are given in SI. Table 2. Results at ꞷB97XD/6-311++G(2d, 2p) are given in main manuscript. Because for appropriate results full range separated ꞷB97XD functional is quite important for calculating optical properties of doped complexes [[51], [52], [53], [54]]. Furthermore, crucial excited states are analyzed using TD-DFT method. To calculate polarizability, below given Equation (4) is applied

(4) *α*_*o*_ = 1/3(*α*_xx_ + *α*_yy_ + *α*_zz_)

The *β*_o_ is calculated by using following Equation (5):

(5) *β*_*o*_ = [*β*_x_^2^ + *β*_y_^2^ + *β*_z_^2^]^1/2^

*β*_x_ = *β*_xxx_ + *β*_xyy_ + *β*_xzz_ *β*_y_ = *β*_yyy_ + *β*_yzz_+ *β*_yxx_ & *β*_z_ = *β*_zzz_+ *β*_zxx_ +*β*_zyy_

Variational trend of dual metals doped C_6_O_6_Li_6_ complexes is examined through the well-known three- and two-level models. Three- and two-level models are described through Equation (6):

(6) *β*˳ ≈ Δμ × *f˳* / ΔE^3^

here, Δμ = Change in the dipole moment of crucial excited state and ground state.

Δ E = Crucial excitation energy

*f˳* = Oscillation strength.

## 3 Results and discussions

**Fig. 1:**
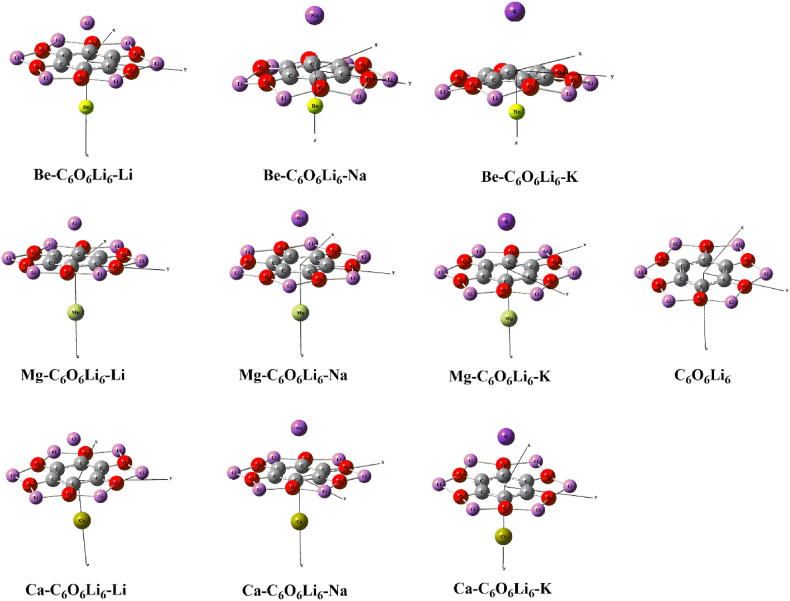
Optimized geometries of isolated and metals doped C_6_O_6_Li_6_ complexes.

The optimized structure of isolated C_6_O_6_Li_6_ is obtained at ωB97XD/6-31+G(d) method and shown in Fig. 1. C_6_O_6_Li_6_ has star like planer structure having *D*_*6h*_ point group symmetry. 1.79 Å, 1.38 Å and 1.41 Å are O–Li, C–O, and C–C bond lengths in C_6_O_6_Li_6_, respectively. These are similar to previously reported values for alk@C_6_O_6_Li_6_ complexes (alk = K, Na, Li) by Mahmood and coworkers [55].

### 3.1 Geometric and thermodynamic properties of dual alkali and alkaline earth metals doped C_6_O_6_Li_6_ electrides

Several dual systems are designed by doping alkali (Li, Na and K) and alkaline earth metals (Be, Mg and Ca) with C_6_O_6_Li_6_. All alk/alkearth earth metals doped (alk-C_6_O_6_Li_6_-alkearth, alk = K, Na, Li, and alkearth = Ca, Mg, Be) complexes have doublet spin state. The charge is zero for isolated C_6_O_6_Li_6_ sheet and all optimized complexes. The isolated C_6_O_6_Li_6_ (singlet, triplet and quintet) sheet and all optimized complexes (doublet, quartet and sextet) are optimized at three lower spin states. It is observed that isolated sheet and the doped complexes are more stable singlet and doublet spin states, respectively. Natural Transition Orbitals. The relative energies of isolated C_6_O_6_Li_6_ and doped complexes at three lower spin states are given in supporting information Table 1 (Table SI. 1). Optimized geometries of pristine C_6_O_6_Li_6_, Be–C_6_O_6_Li_6_–Li, Be–C_6_O_6_Li_6_–Na, Be–C_6_O_6_Li_6_–K, Mg–C_6_O_6_Li_6_–Li, Mg–C_6_O_6_Li_6_–Na, Mg–C_6_O_6_Li_6_–K, Ca–C_6_O_6_Li_6_–Li_6_, Ca–C_6_O_6_Li_6_–Na and Ca–C_6_O_6_Li_6_–K complexes are shown in Fig. 1. After optimization, very minute difference is observed in the bond lengths of C_6_O_6_Li_6_ which are 1.82 Å, 1.36 Å, 1.42 Å for O–Li, C–O and C–C bonds, respectively. Both alkali and alkaline earth metals prefer to reside on the top of the central hexagonal ring of C_6_O_6_Li_6_ which is similar to previously reported alkali metals doped C_6_O_6_Li_6_ complexes [55]. Kosar et al. also reported same behavior for alkaline earth metal on C_24_ nanocage [48]. The interaction distances of Ca/Li, Ca/Na, Ca/K, Mg/Li, Mg/Na, Mg/K, Be/Li, Be/Na and Be/K, from the middle of C_6_O_6_Li_6_ are 2.06/2.79 Å, 2.56/2.74 Å, 2.90/2.73 Å, 2.03/2.84 Å, 2.54/2.69 Å, 2.87/2.68 Å, 2.05/2.47 Å, 2.71/1.65 Å and 3.05/1.63 Å, in Ca–C_6_O_6_Li_6_–Li, Ca–C_6_O_6_Li_6_–Na and Ca–C_6_O_6_Li_6_–K, Be–C_6_O_6_Li_6_–Li, Mg–C_6_O_6_Li_6_–Li, Mg–C_6_O_6_Li_6_–Na, Mg–C_6_O_6_Li_6_–K, Be–C_6_O_6_Li_6_–Na, Be–C_6_O_6_Li_6_–K, complexes, respectively. The internuclear distance of alkali metal is increased with the increase in atomic number. The outcomes are comparable to research report on NLO response of alkali metal doped C_6_O_6_Li_6_ complexes where the distance between both dopant and C_6_O_6_Li_6_ increases with increasing atomic size of alkali metal (from Li to K) [55]. Besides alkali metals, alkaline earth metal doped results are also comparable to Kosar et al. work on nonlinear optical characteristics of alkearth@C_6_O_6_Li_6_ (alkearth = Ca, Mg, Be) complexes where the distance between both dopant and C_6_O_6_Li_6_ increases with increasing atomic size of alkaline earth metals (from Be to Ca, except for Mg where the distance is decreased) [56]. On the other side, the internuclear distance of alkaline earth metal in respective complex is decreased with the increase in atomic number. In case of alkaline earth metals (Ca, Mg, Be), the highest interaction distance (2.84 Å) is obtained for Mg–C_6_O_6_Li_6_–Li and lowest distance (1.63 Å) is calculated for Be–C_6_O_6_Li_6_–K. Upon moving towards alkali metals (K, Na, Li), the highest interaction distance (3.05 Å) is obtained for Be–C_6_O_6_Li_6_–K, and the lowest distance (2.03 Å) is seen for Mg–C_6_O_6_Li_6_–Li (Table 1). In contrary, when the interaction distance between two metals (those are interacting through C_6_O_6_Li_6_ surface) is measured in each of the complex. These interaction distances between alkali and alkaline earth metal in doped complexes ranges from 4.36 Å to 5.64 Å. The lowest interaction distance of 4.36 Å is obtained between Be and Na atoms in Be–C_6_O_6_Li_6_–Na complex. The highest interaction distance (5.64 Å) is seen between Ca and K of Ca–C_6_O_6_Li_6_–K complex.

**Table 1:** The internuclear distance between O and Li (**d**_**O-Li**_ in Å), internuclear distance between C and O (**d**_**C-O**_ in Å), internuclear distance between C and C (**d**_**C-C**_ in Å), the internuclear distance of alkali metal from the center of C_6_O_6_Li_6_ (d_alk_ in Å, Alk = K, Li, Na), internuclear distance of alkaline earth metal from the center of C_6_O_6_Li_6_ (d_alkearth_ in Å, alkearth = Ca, Mg, Be), NBO charges on alkaline earth metals (Q_alkearth_ in |e|), alkali metals (Q_alk_ in |e|), interaction energies (E_int_ in kcal mol^-1^) and vertical ionization energy (VIE in eV) of isolated C_6_O_6_Li_6_ and dual alk-C_6_O_6_Li_6_-alkearth complexes (alk = K, Na, Li, alkearth = Ca, Mg, Be).

Isolated and doped nanosheet	d_O-Li_	d_C-O_	d_C-C_	d_ring-AM_	d_ring-AEM_	Q_AEM_	Q_AM_	E_int_	VIE
C_6_O_6_Li_6_	1.79	1.38	1.41	–	–	–		–	3.04
Be–C_6_O_6_Li_6_–Li	1.82	1.36	1.42	2.05	2.47	−0.02	−0.01	−32.86	3.26
Be–C_6_O_6_Li_6_–Na	1.83	1.34	1.42	2.71	1.65	0.05	0.01	−30.49	2.93
Be–C_6_O_6_Li_6_–K	1.83	1.35	1.42	3.05	1.63	0.06	0.03	−31.13	3.05
Mg–C_6_O_6_Li_6_–Li	1.82	1.36	1.42	2.03	2.84	0.01	0.01	−34.59	3.26
Mg–C_6_O_6_Li_6_–Na	1.81	1.36	1.42	2.54	2.69	0.01	0.01	−29.45	2.93
Mg–C_6_O_6_Li_6_–K	1.81	1.36	1.42	2.87	2.68	0.02	0.03	−29.81	3.21
Ca–C_6_O_6_Li_6_–Li	1.82	1.35	1.42	2.06	2.79	0.05	0.05	−39.99	3.38
Ca–C_6_O_6_Li_6_–Na	1.82	1.36	1.42	2.56	2.74	0.06	0.05	−35.61	3.06
Ca–C_6_O_6_Li_6_–K	1.82	1.36	1.42	2.90	2.73	0.07	0.08	−35.89	3.04

In alkearth-C_6_O_6_Li_6_–Li (alearth = Ca, Mg, Be) electrides, no change in structure of C_6_O_6_Li_6_ is observed after doping. Very small structural variations are seen in alkearth-C_6_O_6_Li_6_–Na and alkearth-C_6_O_6_Li_6_–K (alkearth = Ca, Mg, Be), where Li atom of C_6_O_6_Li_6_ surface is pushed towards alkaline earth metals. Similar structural variations are observed by Ahsan et al. during their studies of alkaline earth metal doped hexaammine complexes [57].

The point group symmetry of isolated C_6_O_6_Li_6 is_ D_6h_. After optimization on the same method their symmetries were changed according to the nature of the metals and their positions on the C_6_O_6_Li_6_ nanosheet. All considered Be–C_6_O_6_Li_6_–Li, Be–C_6_O_6_Li_6_–Na, Be–C_6_O_6_Li_6_–K, Mg–C_6_O_6_Li_6_–Li, Mg–C_6_O_6_Li_6_–Na, Mg–C_6_O_6_Li_6_–K, Ca–C_6_O_6_Li_6_–Li, Ca–C_6_O_6_Li_6_–Na and Ca–C_6_O_6_Li_6_–K complexes have *C*_*6v*_, *C*_*s*_, *C*_*6v*_, *C*_*1*_, *C*_*s*_, *C*_*1*_, *C*_*6v*_, *C*_*1*_ and *C*_*1*_ symmetries, respectively. The change in symmetry represents the change of centrosymmetric to non-centrosymmetric behavior which is ultimate requirement for enhancement of NLO response of any system as reported previously by Kanis et al. [58]. Zyss reported that octupolar molecules with *C*_*2v*_ symmetry are known to have high first hyperpolarizability [59]. We also observed the change in symmetry after doping of alkaline earth metals [56] and alkali [55] on the C_6_O_6_Li_6_.

The interaction energy (E_int_) of a system illustrates thermodynamic stability. The large negative interaction energy reflects exothermicity of the reaction. Thermodynamic stability assures the experimental synthesis and practical application of a complex possible [60]. In this report, we also calculated the interaction energy of all complexes to estimate their thermal stability (Table 1).

The E_int_ of Ca–C_6_O_6_Li_6_–Li, Ca–C_6_O_6_Li_6_–Na, Ca–C_6_O_6_Li_6_–K, Mg–C_6_O_6_Li_6_–Li, Mg–C_6_O_6_Li_6_–Na, Mg–C_6_O_6_Li_6_–K, Be–C_6_O_6_Li_6_–Li, Be–C_6_O_6_Li_6_–Na, Be–C_6_O_6_Li_6_–K are -39.99, -35.61, -35.89, -34.59, -29.45, -29.81, -32.86, -30.49, and -31.13 kcal mol^-1^, respectively. The E_int_ of all complexes illustrates exothermic behavior and indicates their thermodynamic stability. It is also confirmed that metals physiosorbed instead of substitution.

All considered complexes show monotonic decreasing trend of interaction energies with increasing atomic number from Li to Na, and slight increase in E_int_ is observed when we move from Na to K. Interaction energy decreases with increase in atomic size of the alkali metals, and subsequently their stability decreases. Beside interaction distance, atomic sizes of the alkali metals have the major role in affecting the interaction energies. Among alkali metals doping, lithium doped complexes are more stable than sodium and potassium doped complexes in all alkaline/earth metals adsorbed C_6_O_6_Li_6_ complexes. The atomic size of Li is small and interaction distances of Li from aromatic ring (d_ring-Li_) is also shorter which confirms the strong interaction of Li atom with ring and high thermal stability of all Li doped complexes. The outcomes are comparable to research report on NLO response of alkali metal doped C_6_O_6_Li_6_ complexes where the distance between Li and sheet is smaller, followed by K and then Na and subsequently Li doped C_6_O_6_Li_6_ complex is more stable followed by K and then Na doped C_6_O_6_Li_6_ complexes [55]. The same trend of increasing E_int_ is seen for alkearth@C_6_O_6_Li_6_ (alkearth = Ca, Mg, Be) complexes [56].

Overall, Ca–C_6_O_6_Li_6_–Li complex is the most stable based on its highest interaction energy (-39.99 kcal mol^-1^). The exceptional stability of Ca–C_6_O_6_Li_6_–Li is correlated with the average interaction distance of both alkali (*vide supra*) and alkaline earth metals from the surface. As the atomic size of the alkaline earth metal increases their ionization potential also increases which is responsible for strong interaction with C_6_O_6_Li_6_ surface. Other two Ca doped complexes are also more stable than Be and Mg doped complexes.

**Fig. 2:**
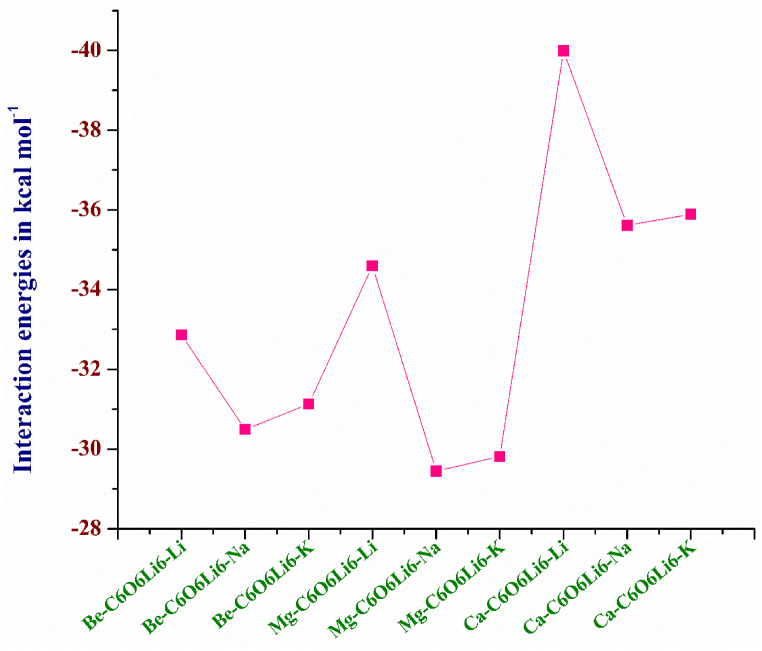
Graphical representation of interaction energies of alk-C_6_O_6_Li_6_-alkearth electrides (alk = K, Na, Li and alkearth = Ca, Mg, Be).

The increasing interaction energy trend of Be series is as: Be–C_6_O_6_Li_6_–Li > Be–C_6_O_6_Li_6_–K > Be–C_6_O_6_Li_6_–Na (see Fig. 2). The very similar increasing interaction energy trend of Mg series is as: Mg–C_6_O_6_Li_6_–Li > Mg–C_6_O_6_Li_6_–K > Mg–C_6_O_6_Li_6_–Na. The increasing E_int_ for Ca series is as: Ca–C_6_O_6_Li_6_–Li > Ca–C_6_O_6_Li_6_–Na > Ca–C_6_O_6_Li_6_–K. Similar results are reported in previous study of alkaline earth metal doped complexes [57]. Literature reveals that the interaction of any metal atom with any surface depends upon the properties of that surface as well as the stable spin state of that particular doped structure [61]. In our complexes, C_6_O_6_Li_6_ compound has flexibility to absorb electron donor alkali and alkaline earth metals which formed strong interaction with this surface at doublet spin state.

### 3.2 FMOs analysis and electride properties of dual alkali/alkaline earth metals doped C_6_O_6_Li_6_ complexes

For obtaining the qualitative analysis of electronic properties and nature of dual alk/alkearth metals doped complexes, FMOs analysis is performed. The energies of LUMOs (E_L_), HOMOs (E_H_), and respective energy gaps (E_H-L_) of isolated C_6_O_6_Li_6_ and alk/alkearth doped complexes are given in Table 2. The calculated HOMO-LUMO energy gap (E_H-L_) of isolated C_6_O_6_Li_6_ is 4.63 eV. After decorating of alk/alkearth on the surface resulted in variation of respective E_H-L_ from 2.94 eV to 3.40 eV. The VIE results of our current work is more than the VIE values reported previously for alk-C_6_O_6_Li_6_ complexes. These results indicate more stability of dual doped complexes [55]. The lowest gaps are seen for Be–C_6_O_6_Li_6_–K and Mg–C_6_O_6_Li_6_–K 2.94 eV. The reason for the reduction in energy gap is related to change in the E_HOMO_ and E_LUMO_ in all doped complexes. But significant increase in the E_HOMO_ and decrease in the E_LUMO_ occur of Be–C_6_O_6_Li_6_–K and Mg–C_6_O_6_Li_6_–K which causes large decrease in E_H-L_. For Be–C_6_O_6_Li_6_–K complex, the E_HOMO_, E_LUMO_ and E_H-L_ are -2.93, 0.03, and 2.94 eV, respectively. For Mg–C_6_O_6_Li_6_–K complex, the E_HOMO_, E_LUMO_ and E_H-L_ are -2.93, 0.07, and 2.94 eV, respectively. The highest gap is seen for Ca–C_6_O_6_Li_6_–Na complex. In the case of Ca–C_6_O_6_Li_6_–K complex, the E_HOMO_, E_LUMO_ and E_H-L_ are -3.38, 0.01, and 3.40 eV, respectively. The lower HOMO-LUMO gaps indicate that these complexes may have enhanced NLO response as observed by Fonseca et al. in their work on NLO response of chromophores [62].

**Table 2:** E_L-H_ in eV, the energies of LUMO (E_LUMO_), the energies of HOMO (E_HOMO_), the first hyperpolarizability (*β*_o_), *β*_*Z*_ under three-level model, polarizability (*α*ₒ) in au, and dipole moment (*μ* in Debye) of isolated C_6_O_6_Li_6_ and Be–C_6_O_6_Li_6_-alk (alk = K, Na, Li) electrides.

Isolated and doped C_6_O_6_Li_6_	E_L-H_	E_LUMO_	E_HOMO_	*β*_o_	*β*_Z_	*α*ₒ	*μ*
**C**_**6**_**O**_**6**_**Li**_**6**_	4.63	0.24	−4.39	2.89	… …..	136.62	0.00
**Be–C**_**6**_**O**_**6**_**Li**_**6**_**–Li**	3.11	0.06	−3.04	1.57 × 10^4^	3.47 × 10^4^	544.51	3.36
**Be–C**_**6**_**O**_**6**_**Li**_**6**_**–Na**	3.31	0.06	−3.26	1.89 × 10^4^	−168.48	508.12	4.63
**Be–C**_**6**_**O**_**6**_**Li**_**6**_**–K**	2.94	0.01	−2.93	1.55 × 10^4^	−523.12	775.92	6.33
**Mg–C**_**6**_**O**_**6**_**Li**_**6**_**–Li**	3.11	0.06	−3.05	1.43 × 10^4^	3.64 × 10^4^	586.46	2.86
**Mg–C**_**6**_**O**_**6**_**Li**_**6**_**–Na**	3.33	0.07	−3.26	1.03 × 10^4^	3.37 × 10^4^	529.95	4.16
**Mg–C**_**6**_**O**_**6**_**Li**_**6**_**–K**	2.94	0.01	−2.93	1.68 × 10^5^	−423.28	570.54	0.10
**Ca–C**_**6**_**O**_**6**_**Li**_**6**_**–Li**	3.21	−0.01	−3.21	1.39 × 10^4^	3.82 × 10^4^	631.73	0.10
**Ca–C**_**6**_**O**_**6**_**Li**_**6**_**–Na**	3.40	0.01	−3.38	7.49 × 10^3^	2.25 × 10^4^	629.28	0.11
**Ca–C**_**6**_**O**_**6**_**Li**_**6**_**–K**	3.00	−0.05	−3.06	1.14 × 10^5^	5.78 × 10^4^	604.07	1.46

**Fig. 3:**
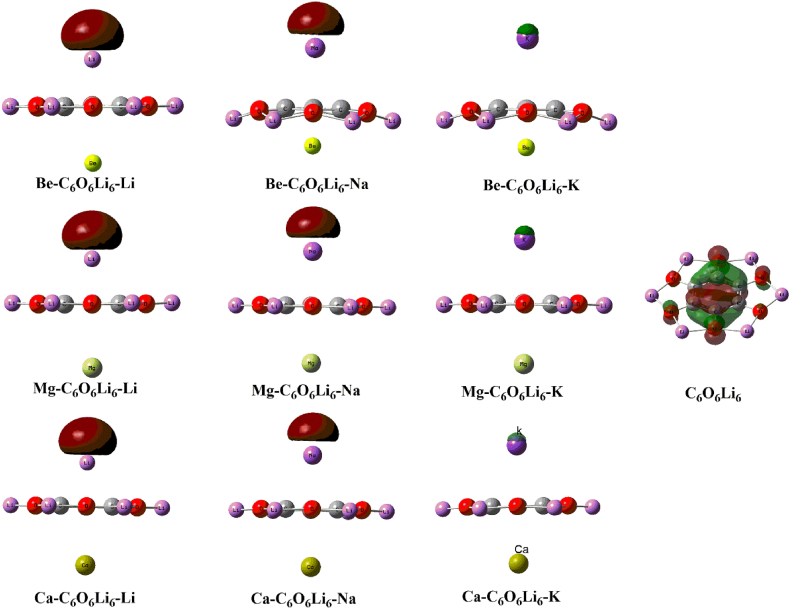
Graphical representation for HOMOs of isolated C_6_O_6_Li_6_ and dual alkali/alkaline earth (alk/alkearth) metals doped C_6_O_6_Li_6_ electrides (isovalue = 0.05).

The conducting properties (insulator, semiconductor and metallic) of a system is reflected to E_H-L_ values, as observed previously [63]. The gaps in the current report justify semiconducting behavior (gap is more than 1 eV) of electrides. The reduction in E_H-L_ after dual metals doping is because of new HOMOs generation at increased energy due to electronic charge from metals to C_6_O_6_Li_6_ electride surface. In the previous literature such shifting is observed from metals towards nanosurface [64,65]. For isolated C_6_O_6_Li_6_ and dual metals doped electrides, the isodensities surface of the frontier molecular orbitals (HOMOs and LUMOs) are generated and their structures are given in Fig. 3 and SI. Fig. 2. The molecular orbitals isodensities distribution of HOMOs are carefully analyzed and it is observed that densities occupy the free space in some of the complexes. None of the HOMO densities are present on any atom. The presence of such type of HOMOs densities in free space of complexes prove their electrides properties. The electides properties of Li and Na doped C_6_O_6_Li_6_ complex are similar to our previous work on alk-C_6_O_6_Li_6_ complexes where Na and Li doped C_6_O_6_Li_6_ complexes show electrides properties [55]. Exceptional behavior is observed for alkearth-C_6_O_6_Li_6_–K, (alkearth = Ca, Mg, Be). Here, the highest occupied molecular orbitals (HOMOs) isodensities are localized on the alkali metals (Li, Na) in alk-C_6_O_6_Li_6_-alkearth (Alk = K, Na, Li, and alkearth = Ca, Mg, Be) and have no electrides properties. Thus, these latter species (alk-C_6_O_6_Li_6_-alkearth (Alk = K, Na, Li, and alkearth = Ca, Mg, Be)) act as excess electron species. Previously, Mahmood and coworkers also seen electrides behavior in doping of alkali metals on C_6_O_6_Li_6_ [55] again we observed electride properties in our current dual metal doped complexes. Zheng and coworkers also seen such electrides properties for dual alkali metal doped on three-ring Janus face C_13_H_10_F_12_ surfaces [11]. Ma et al. designed Li atoms doped B_20_H_26_ electrides and their results depict that electrides show significant NLO response [35].

### 3.3 Charge transfer analysis of dual alkali/alkaline earth metals (alk/alkearth) doped C_6_O_6_Li_6_ electrides

For further explanation of electronic properties of alk-C_6_O_6_Li_6_-alkearth electrides (alk-C_6_O_6_Li_6_-alkearth (Alk = K, Na, Li, and alkearth = Ca, Mg, Be)), we performed charge transfer by using natural bond orbital method of all electrides and the NBOs graphics are given in supplementary information (SI. Fig. 3). The graphics of NBOs showed similar patterns of orbitals densities as we seen in FMOs in Fig. 3. NBO analysis revealed that all alkali and alkaline earth metals have positive charges. NBO analysis shows that charge is transferred from the alkali metals and alkaline earth metals to C_6_O_6_Li_6_ in these electrides. The charges on alkali metals ranges from 0.010 to 0.076 |e| whereas charges on alkaline earth metals are between 0.011 and 0.071 |e| in dual alk/alkearth doped C_6_O_6_Li_6_ electrides. The NBO charges on Ca (0.076 |e|) and K metals (0.071 |e|) are the highest as compared to all alk/alkearth doped C_6_O_6_Li_6_ electrides. The NBO charge on Be/Na, Be/K, Mg/Li, Mg/Na, Mg/K, Ca/Li and Ca/Na are 0.049/0.013, 0.060/0.032, 0.007/0.010, 0.011/0.010, 0.015/0.028, 0.054/0.053 and 0.059/0.046 |e|, respectively. The intensity of charge transfer depends upon the lower ionization potential and large atomic sizes of K and Ca across the column of the periodic table [66,67]. This may be is the reason for easily loss of electrons in our current work. Exceptional behavior is seen for Be and Li metals in Be–C_6_O_6_Li_6_–Li electrides those have negative charge. The NBO charges are -0.018 and -0.001 |e| on Be and Li metals of Be–C_6_O_6_Li_6_–Li electrides. The negative charge on metal is due to the localization of electronic density closer to the metal center due to which the small negative charge is observed on the respective metal [22,[68], [69], [70]].

The vertical ionization energies (VIEs) values of designed electrides are between 2.93 and 3.38 eV. The larger VIEs values also justify the high thermal stability of these electrides according to already reported literature [[71], [72], [73]]. The VIEs values of all electrides are smaller than 3.89 eV VIE of Cesium which illustrates the presence of diffuse excess electrons in these electrides [13,74].

The C_6_O_6_Li_6_ has a zero dipole moment due to centrosymmteric nature. When alk/alkearth metals are doped on this surface, the charge transfer occurs between these species. Which causes variation in the symmetry of C_6_O_6_Li_6_ and ultimately increases the *μ*_o_ of designed electrides. The *μ*_o_ of Be–C_6_O_6_Li_6_–Li, Be–C_6_O_6_Li_6_–Na, Be–C_6_O_6_Li_6_–K, Mg–C_6_O_6_Li_6_–Li, Mg–C_6_O_6_Li_6_–Na, Mg–C_6_O_6_Li_6_–K, Ca–C_6_O_6_Li_6_–Li, Ca–C_6_O_6_Li_6_–Na and Ca–C_6_O_6_Li_6_–K electrides is 3.36 D, 4.63 D, 6.33 D, 2.86 D, 4.16 D, 0.10 D, 0.10 D, 0.11 D, and 1.46 D, respectively (Table 2). The highest dipole moment is seen for Be–C_6_O_6_Li_6_–K is 6.33 D. The charge separation between surface (C_6_O_6_Li_6_) and alk/alkearth metals (K, Na, Li/Ca, Mg, Be) along with interatomic distance between both of these result in increase of the *μ*_o_ of these electrides than the isolated C_6_O_6_Li_6_.

### 3.4 Polarizaibility (α_o_) and the first hyperpolarizability (β_o_) analyses of dual alkali/alkaline earth metals (alk/alkearth) doped C_6_O_6_Li_6_ electrides

The NLO response is surely enhanced with the introduction of excess electrons in a complex. All required parameters of respective newly designed electrides are calculated for estimation of NLO response. The *α*_o_ and *β*_o_ are calculated at the same ꞷB97XD, CAM-B3LYP, and LC-BLYP functional and Pople’s basis set (6-311++G(2d, 2p)) for appropriate estimation of nonlinear response. The results at CAM-B3LYP, and LC-BLYP functional and Pople’s basis set (6-311++G(2d, 2p)) are given in SI. Table 2 while the results at ꞷB97XD/6-311++G(2d, 2p) are given in Table 2 of the main manuscript.

The *α*_o_ of newly designed excess electrons electrides are investigated to estimate linear optical response. The *α*_o_ of alk-C_6_O_6_Li_6_-alkearth (alkearth = Ca, Mg, Be and alk = K, Na, Li) electrides is in the range of 508–775 au, which is very high as compared to the isolated C_6_O_6_Li_6_ (137 au). The nonmonotonic increasing *α*_o_ trend is observed for all dual alk/alkearth doped electrides. The trend of *α*_o_ is as follows; Be–C_6_O_6_Li_6_–K (775.92 au) > Ca–C_6_O_6_Li_6_–Li (631.73 au) > Ca–C_6_O_6_Li_6_–Na (629.28 au) > Ca–C_6_O_6_Li_6_–K (604.07 au) > Mg–C_6_O_6_Li_6_–Li (586.46 au) > Mg–C_6_O_6_Li_6_–K (570.54 au) > Be–C_6_O_6_Li_6_–Li (544.51 au) > Mg–C_6_O_6_Li_6_–Na (529.95 au) > Be–C_6_O_6_Li_6_–Na (508.12 au). Results of polarization are quite comparable to the interatomic distance of Be metal which is lower (1.63 Å) and the largest distance (3.05 Å) is observed for K in this complex, followed by other metals. Subsequently, this lower internuclear distance and charge transfer causes polarization (*α*_o_) changes in these electrides.

The *β*_o_ of C_6_O_6_Li_6_ is considerably increased after alk/alkearth metals are introduced. The *β*_o_ value of pristine system (C_6_O_6_Li_6_) is 2.89 au. As a result of dual alk/alkearth metals doping, the remarkable NLO response of (alk-C_6_O_6_Li_6_-alkearth, alkearth = Ca, Mg, Be and alk = K, Na, Li) electrides is observed and the static first hyperpolarizabilities (*β*_o_) are tremendously increased. The *β*_o_ of electrides are in the range of 7.49 × 10^3^–1.68 × 10^5^ au. The highest *β*_o_ is observed for Mg–C_6_O_6_Li_6_–K which is 1.68 × 10^5^ au while the lowest value (7.49 × 10^3^ a. u) is computed for Ca–C_6_O_6_Li_6_–Na. The results revealed the monotonic increasing trend of *β*_o_ from Be–C_6_O_6_Li_6_–K to Mg–C_6_O_6_Li_6_–K, followed by lower *β*_o_ of Ca–C_6_O_6_Li_6_–K. The *β*_o_ results are similar to our previous work on NLO response of alkali metal doped C_6_O_6_Li_6_ complexes where K doped C_6_O_6_Li_6_ complex had the highest *β*_o_ of 2.60 × 10^5^ au among all selected alkali metal doped C_6_O_6_Li_6_ complexes [55]. The trend of increasing is noticed for alkaline earth metal doped C_6_O_6_Li_6_ complexes where Mg@C_6_O_6_Li_6_ complex has the highest *β*_o_ (7.11 × 10^6^ au) followed by other Ca and Be doped complexes [56]. The *β*_o_ values of designed alk/alkearth metals doped electrides are influenced by various factors, vertical ionization potential, interaction distance of metal atom and charge transfer on the doped metal atoms compared to isolated C_6_O_6_Li_6_. The *β*_o_ value obtained for Cr@C_6_O_6_Li_6_ is 1.68 × 10^5^ au which is higher than previously reported for M@calix [4]pyrrole (M = Li, Na and K). The *β*_o_ values increases in order *i.e.* K@calix [4]pyrrole (1.7 × 10^4^ au) > Na@calix [4]pyrrole (7.5 × 10^3^ au) > Li@calix [4]pyrrole (7.3 × 10^3^ au). In this regard, another example is A_3_@GDY (GDY = graphdiyne and A = Li, Na and K) where hyperpolarizability shows an increasing trend by the increase in atomic radii of the alkali atoms from Li to K. The highest hyperpolarizability of 1.61 × 10^5^ au is obtained for K_3_@GDY [85]. The intermolecular charge transfer interactions are responsible for NLO response as discussed by Zyss and Berthier in a work on NLO response of urea [75]***.***

### 3.5 Three level model of dual alkali and alkaline earth metals doped C_6_O_6_Li_6_ electrides

The internal parameters responsible for variational *β*_o_ values of these electrides are evaluated by implementing three and two level models (***β***_TLM_) with the use of MultiWfn software [76]. The data of three level model is given in main manuscript while two level model results are given in SI. Table 3. Following is the mathematical form of two-level model.

7 ***β***_TLM_ ≈ Δμ × *f*_o_/ΔE^3^

Above equation reveals that crucial excitation energies are inversely related to *β*_TLM_ but it has linear increment with oscillation strength (*f*_o_) and change in dipole moment (Δ*μ*). The ***β***_TLM_ results depicts that the change of excitation energy [77] has the key role for the increase in the hyperpolarizability values of these electrides. The excitation energies of Be–C_6_O_6_Li_6_–Li@, Be–C_6_O_6_Li_6_–Na, Be–C_6_O_6_Li_6_–K for the first excited state are 0.93 eV, 1.73 eV and 0.88 eV, respectively. In Mg–C_6_O_6_Li_6_–Li, Mg–C_6_O_6_Li_6_–Na and Mg–C_6_O_6_Li_6_–K, the excitation energies are 0.94, 1.11, 0.76 eV, respectively. In Ca–C_6_O_6_Li_6_–Li, Ca–C_6_O_6_Li_6_–Na and Ca–C_6_O_6_Li_6_–K, the excitation energies are 2.08, 2.00 and 0.79 eV, respectively. The increasing trend in crucial excitation energies of Mg doped electrides is comparable to that of the first hyperpolarizability values in each series of alk-C_6_O_6_Li_6_ (alk = K, Na, Li). *β*_o_ of Mg–C_6_O_6_Li_6_–K is 1.68 × 10^5^ au which has excitation energy of 0.76 eV. In current scenario, if the excitation energy is smaller for an electrides then *β*_o_ for this complex is also high.

**Table 3:** The variation in dipole moment between ground and crucial excited state (Δ*μ* in Debye), oscillating strength (ƒₒ), change in excitation energy (ΔE in eV), hyperpolariziability at three level model (*β*_TLM_**)** of 1st and 2nd excited states, the static first hyperpolariizaibility (*β*_o_) and maximum wavelength (*λ*_max_ in nm) of isolated C_6_O_6_Li_6_ and Mg–C_6_O_6_Li_6_-alk (alk = K, Na, Li) electrides.

Parameters	Excited state 1			Excited state 2			*β*_TLM_	*β*_o_	*λ*_max_
	Δ*μ*	ΔE	*ƒ*_o_	Δ*μ*	ΔE	*ƒ*_o_			
**C**_**6**_**O**_**6**_**Li**_**6**_	0.01	2.42	0.03	0.01	2.42	0.04	1.22	2.89	515
**Be–C**_**6**_**O**_**6**_**Li**_**6**_**–Li**	−2.48	0.93	0.000090	−2.47	0.93	0.000026	−65.23	1.57 × 10^4^	718
**Be–C**_**6**_**O**_**6**_**Li**_**6**_**–Na**	1.35	1.73	0.000024	1.44	1.73	0.000044	3.33	1.89 × 10^4^	841
**Be–C**_**6**_**O**_**6**_**Li**_**6**_**–K**	2.70	0.88	0.000002	2.70	0.88	0.000136	100.70	1.55 × 10^4^	792
**Mg–C**_**6**_**O**_**6**_**Li**_**6**_**–Li**	−2.66	0.94	0.000002	−2.67	0.94	0.000004	−3.49	1.43 × 10^4^	783
**Mg–C**_**6**_**O**_**6**_**Li**_**6**_**–Na**	−2.05	1.11	0.000000	−2.05	1.11	0.000000	−0.05	1.03 × 10^4^	729
**Mg–C**_**6**_**O**_**6**_**Li**_**6**_**–K**	2.17	0.76	0.000000	2.17	0.77	0.000001	1.28	1.68 × 10^5^	1622
**Ca–C**_**6**_**O**_**6**_**Li**_**6**_**–Li**	1.17	2.08	0.000007	0.64	2.08	0.000002	0.16	1.39 × 10^4^	743
**Ca–C**_**6**_**O**_**6**_**Li**_**6**_**–Na**	−0.72	2.00	0.000000	−0.32	2.00	0.001392	−10.08	7.49 × 10^3^	701
**Ca–C**_**6**_**O**_**6**_**Li**_**6**_**–K**	−0.13	0.79	0.164759	−0.10	0.79	0.031204	−10281.57	1.14 × 10^5^	828

We see that beside excitation energy, other factors which influence the values of hyperpolarizability are oscillation strength (*f*_o_) and dipole moment (Δ*μ*) of that particular electrides. The increasing trend of *f*_o_ and Δ*μ* are opposite to that of hyperpolarizability values for all alk-C_6_O_6_Li_6_-alkearth (alkearth = Ca, Mg, Be, alk = K, Na, Li) electrides. Exceptional behavior is seen for oscillating strength values of Ca doped complexes where the trend of increasing oscillating strength is similar to hyperpolarizability values. Thus, excitation energy of the first excited state is the main factor responsible for the variation in hyperpolarizability of each complex.

The excitation energies of Be–C_6_O_6_Li_6_–Li, Be–C_6_O_6_Li_6_–Na, Be–C_6_O_6_Li_6_–K for the second excited state are 0.93, 1.73 and 0.88 eV, respectively. In Mg–C_6_O_6_Li_6_–Li, Mg–C_6_O_6_Li_6_–Na and Mg–C_6_O_6_Li_6_–K, the excitation energies are 0.94, 1.11, 0.77 eV, respectively. In Ca–C_6_O_6_Li_6_–Li, Ca–C_6_O_6_Li_6_–Na and Ca–C_6_O_6_Li_6_–K, the excitation energies are 2.08 eV, 2.00 eV and 0.79 eV, respectively. The increasing excitation energies trend of Mg doped electrides is comparable to that of the first hyperpolarizability values in each series of alkali metals@C_6_O_6_Li_6_ (alk = K, Na, K). The *β*_o_ of Mg–C_6_O_6_Li_6_–K is 1.68 × 10^5^ au which has excitation energy of 0.77 eV. Here, again it is seen that if the ΔE is smaller for an electrides than *β*_o_ for this complex is also high. Table 3 show that trend in the increasing *f*_o_ and Δ*μ* are opposite to that of hyperpolarizability values for all alk-C_6_O_6_Li_6_-alkearth (alkearth = Ca, Mg, Be and alk = K, Na, Li) electrides. Exceptional behavior is seen for oscillating strength values of Mg doped complexes where the trend of increasing oscillating strength is similar to hyperpolarizability values. Conclusively, the excitation energies of the second excited state are the main contributor to the hyperpolarizability results of all electrides. The increasing trend of *β*_TLM_ values of both first and second excited states are opposite to the *β*_o_ values for all complexes. But the Ca–C_6_O_6_Li_6_–K complex has the higher value of *β*_o_ which halos has the highest *β*_TLM_ value.

We also calculated *β*_z_ values and observed similar variational trend of *β*_z_ values as we seen in *β*_o_ values for dual alk/alkearth metals doped C_6_O_6_Li_6_ electrides. Sunaina et al. observed similar comparable trend of *β*_o_ and *β*_z_ values for alkali metals doped C_6_O_6_Li_6_ organometallic [55].

### 3.6 TD-DFT calculation of dual alkali and alkaline earth metals doped C_6_O_6_Li_6_ electrides

**Fig. 4:**
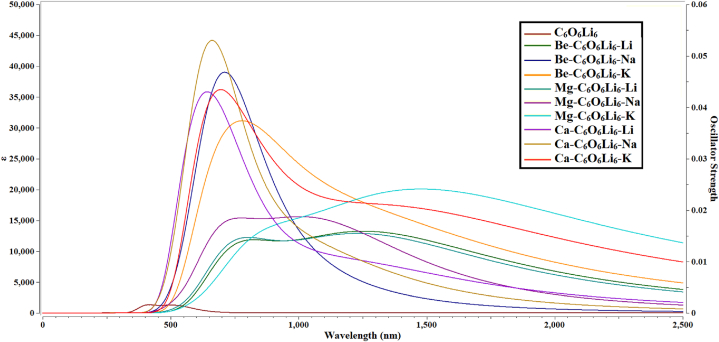
Ultra-violet-visible (UV–Vis) spectra of isolated and alk-C_6_O_6_Li_6_-alkearth electrides (alkearth = Ca, Mg, Be and alk = K, Na, Li).

As discussed earlier, nonlinear optical materials having high *β*_o_ are used in second harmonic generation for doubling of frequency [60,78]. To check the UV transparency of these electrides, we performed TD-DFT analysis of the isolated C_6_O_6_Li_6_ and alk-C_6_O_6_Li_6_-alkearth electrides. The spectra of isolated and doped electrides ((alk-C_6_O_6_Li_6_-alkearth, alkearth = Ca, Mg, Be and alk = K, Na, Li)) are shown in Fig. 4. The absorption maxima (*λ*_max_) of alk-C_6_O_6_Li_6_-alkearth (alkearth = Ca, Mg, Be and alk = K, Na, Li) ranges from 701 to 1622 nm, the highest value (1622 nm) is observed for Mg–C_6_O_6_Li_6_–K and the lowest value (701 nm) is observed for Ca–C_6_O_6_Li_6_–Na electrides. The outcomes of UV–Vis analysis are comparable to the research report on NLO response of alkali metal doped C_6_O_6_Li_6_ complexes where K doped C_6_O_6_Li_6_ complex had the highest *λ*_max_ among all selected alkali metal doped C_6_O_6_Li_6_ complexes [55]. The similar trend of increasing *λ*_max_ is noticed for alkaline earth metal doped C_6_O_6_Li_6_ complexes where Be–C_6_O_6_Li_6_ complex has the highest *λ*_max_ followed by other Ca and Mg doped complexes [56]. Thus, alk-C_6_O_6_Li_6_-alkearth (alkearth = Ca, Mg, Be and alk = K, Na, Li) electrides show red shifts in comparesion to isolated C_6_O_6_Li_6_ surface (*λ*_max_ = 519 nm). The red shift behavior of alk/alkearth metals is comparable to alkearth@complexes (alkearth = Ca, Mg, Be) [79] and alkali metals [80] doped complexes which justify our results. Transparency of alk-C_6_O_6_Li_6_-alkearth electrides in deep UV region notify them as potential candidates for use in UV-NLO materials. Zyss and coworkers observed that non-centrosymmetric octupolar molecules show higher NLO response along with transparency [81] as we observed for our octupolar complexes. Natural Transition Orbitals have been generated for all of the complexes; their figures have been added in supplementary information SI Fig. 4. These results justify the movement of electronic density to free spaces between metals and nano surface in case of Li and doped complexes and confirmed their electrides properties.

The π to π* transition is more prominent in isolated C_6_O_6_Li_6_. The wavelength 515 nm also justify this electronic excitation type which is in the range of 200–700 nm reported for π to π* transition in literature [82]. When alkali and alkaline earth metal are doped on C_6_O_6_Li_6_ surface, a charge transfer electronic excitation is seen where the densities are present on alkali and alkaline earth metal in higher excitation states. In the case of the lowest excited states, transition orbitals densities on central aromatic ring of the nanosheet in each of the complex show π to π* transition with in the C_6_O_6_Li_6_ surface.

## 4 Conclusion

Linear and NLO response of dual alk/alkearth metals doped C_6_O_6_Li_6_ (alk-C_6_O_6_Li_6_-alkearth, alkearth = Ca, Mg, Be and alk = K, Na, Li) electrides is studied. Metals act as source of excess electrons which introduced nonlinear optical properties. The designed alk/alkearth metals doped C_6_O_6_Li_6_ electrides (alk-C_6_O_6_Li_6_-alkearth, alkearth = Ca, Mg, Be and alk = K, Na, Li) have interaction energies between -39.99 and -29.45 kcal mol^-1^ which demonstrates their thermal feasibility. The larger VIEs values of 2.93–3.38 eV also justify the high electronic stability of these electrides. Natural bond orbital charge analysis is implemented to evaluate the charge transfer from C_6_O_6_Li_6_ to alkali and alkaline earth metal and *vice versa*. Furthermore, frontier molecular orbitals (FMOs) analysis validated electronic density distributions in these electrides.. The smaller HOMO-LUMO gap and moderate VIEs exhibited by new electrides depicts their semiconducting behavior. The highest *β*_o_ observed for Mg–C_6_O_6_Li_6_–K is 1.68 × 10^5^ au. The remarkable NLO response based on higher *β*_o_ values of these electrides make them very potent to be used in electronics and lasers technologies. The increasing trend in crucial excitation energies of doped electrides is comparable to that of the first hyperpolarizability values in each series of dual alk-C_6_O_6_Li_6_-alkearth electrides (alk = K, Na, Li and alkearth = Ca, Mg, Be) calculated through three level model. Similarly, the variational trend of *β*_z_ values is also comparable to *β*_o_ values. Transparency of alk-C_6_O_6_Li_6_-alkearth electrides in deep UV region notify them as potential candidates for use in UV-NLO materials.

## Author contribution statement

Naveen Kosar, Sunaina Wajid, Mazhar Amjad Gilani: Performed the experiments; Analyzed and interpreted the data; Wrote the paper.

Nur Hazimah Binti Zainal Arfan, Malai Haniti Sheikh Abdul Hamid, Muhammad Imran: Analyzed and interpreted the data; Contributed reagents, materials, analysis tools or data; Wrote the paper.

Khurshid Ayub, Nadeem S. Sheikh, Tariq Mahmood: Conceived and designed the experiments; Analyzed and interpreted the data; Wrote the paper.

## Declaration of competing interest

The authors declare that they have no known competing financial interests or personal relationships that could have appeared to influence the work reported in this paper.
