# Supplementary material for: Giant NLO response and deep ultraviolet transparency of dual (alkali/alkaline earth) metals doped C6O6Li6 electrides
Source: Heliyon. 2023 Jul 13;9(8):e18264. doi: 10.1016/j.heliyon.2023.e18264 (PMC10391932; doi:10.1016/j.heliyon.2023.e18264)
Supplement: Multimedia component 1 [file mmc1.docx]

**Supplementary Information**

**SI. Table S1:** Interaction energies of (E_int_ in kcal mol^-1^) of pure C_6_O_6_Li_6_ and A/B@C_6_O_6_Li_6_ (A = Be, Mg, Ca, and B = Li, Na, K) complexes at lower spin states .

| **Pure and doped complexes** | **ꙌB97XD/6-311++G(2d,2p)** | | |
| --- | --- | --- | --- |
| **Spin states** | **0, 1** | **0, 3** | **0, 5** |
| **Pure C_6_O_6_Li_6_** | 0.00 | 52.00 | 125.04 |
| **Spin states** | **0, 2** | **0, 4** | **0, 6** |
| **Be/Li@C_6_O_6_Li_6_** | 0.00 | 49.98 | 124.29 |
| **Be/Na@C_6_O_6_Li_6_** | 0.00 | 32.67 | 127.45 |
| **Be/K@C_6_O_6_Li_6_** | 0.00 | 32.50 | 127.59 |
| **Mg/Li@C_6_O_6_Li_6_** | 0.00 | 51.65 | 125.76 |
| **Mg/Na@C_6_O_6_Li_6_** | 0.00 | 49.50 | 121.17 |
| **Mg/K@C_6_O_6_Li_6_** | 0.00 | 49.13 | 120.84 |
| **Ca/Li@C_6_O_6_Li_6_** | 0.00 | 32.54 | 109.92 |
| **Ca/Na@C_6_O_6_Li_6_** | 0.00 | 31.55 | 105.15 |
| **Ca/K@C_6_O_6_Li_6_** | 0.00 | 31.44 | 104.65 |

**SI. Table 2:** Dipole moment (*µ* in Debye), polarizability (*α*ₒ in au), and the first hyperpolarizability (*β*_o_ in au) of pure C_6_O_6_Li_6_ and A/B@C_6_O_6_Li_6_ (A = Be, Mg, Ca, and B = Li, Na, K) complexes.

| **Pure and doped complexes** | **CAM-B3LYP** | | | **LC-BLYP** | | |
| --- | --- | --- | --- | --- | --- | --- |
|  | **6-311++g(2d,2p)** | | | | | |
|  | ***µ*** | ***α*ₒ** | ***β*_o_** | ***µ*** | ***α*ₒ** | ***β*_o_** |
| **Pure C_6_O_6_Li_6_** | 0.00 | 109.71 | 160.11 | 0.00 | 123.71 | 180.20 |
| **Be/Li@C_6_O_6_Li_6_** | 2.35 | 624.18 | 1.24×10^5^ | 3.22 | 485.36 | 1.31×10^4^ |
| **Be/Na@C_6_O_6_Li_6_** | 3.98 | 478.36 | 2.15×10^4^ | 3.84 | 417.85 | 1.58×10^4^ |
| **Be/K@C_6_O_6_Li_6_** | 5.11 | 718.92 | 4.52×10^4^ | 5.36 | 660.90 | 9.01×10^3^ |
| **Mg/Li@C_6_O_6_Li_6_** | 1.88 | 663.53 | 1.22×10^5^ | 2.66 | 520.71 | 1.46×10^4^ |
| **Mg/Na@C_6_O_6_Li_6_** | 1.58 | 490.41 | 1.53×10^4^ | 1.98 | 438.06 | 8.86×10^3^ |
| **Mg/K@C_6_O_6_Li_6_** | 2.20 | 868.33 | 1.22×10^5^ | 3.17 | 789.41 | 1.08×10^5^ |
| **Ca/Li@C_6_O_6_Li_6_** | 0.36 | 673.95 | 4.54×10^4^ | 0.16 | 572.72 | 5.35×10^2^ |
| **Ca/Na@C_6_O_6_Li_6_** | 0.81 | 601.04 | 7.49×10^3^ | 0.91 | 547.22 | 9.278×10^3^ |
| **Ca/K@C_6_O_6_Li_6_** | 0.05 | 903.46 | 1.17×10^5^ | 0.36 | 838.13 | 5.83×10^4^ |


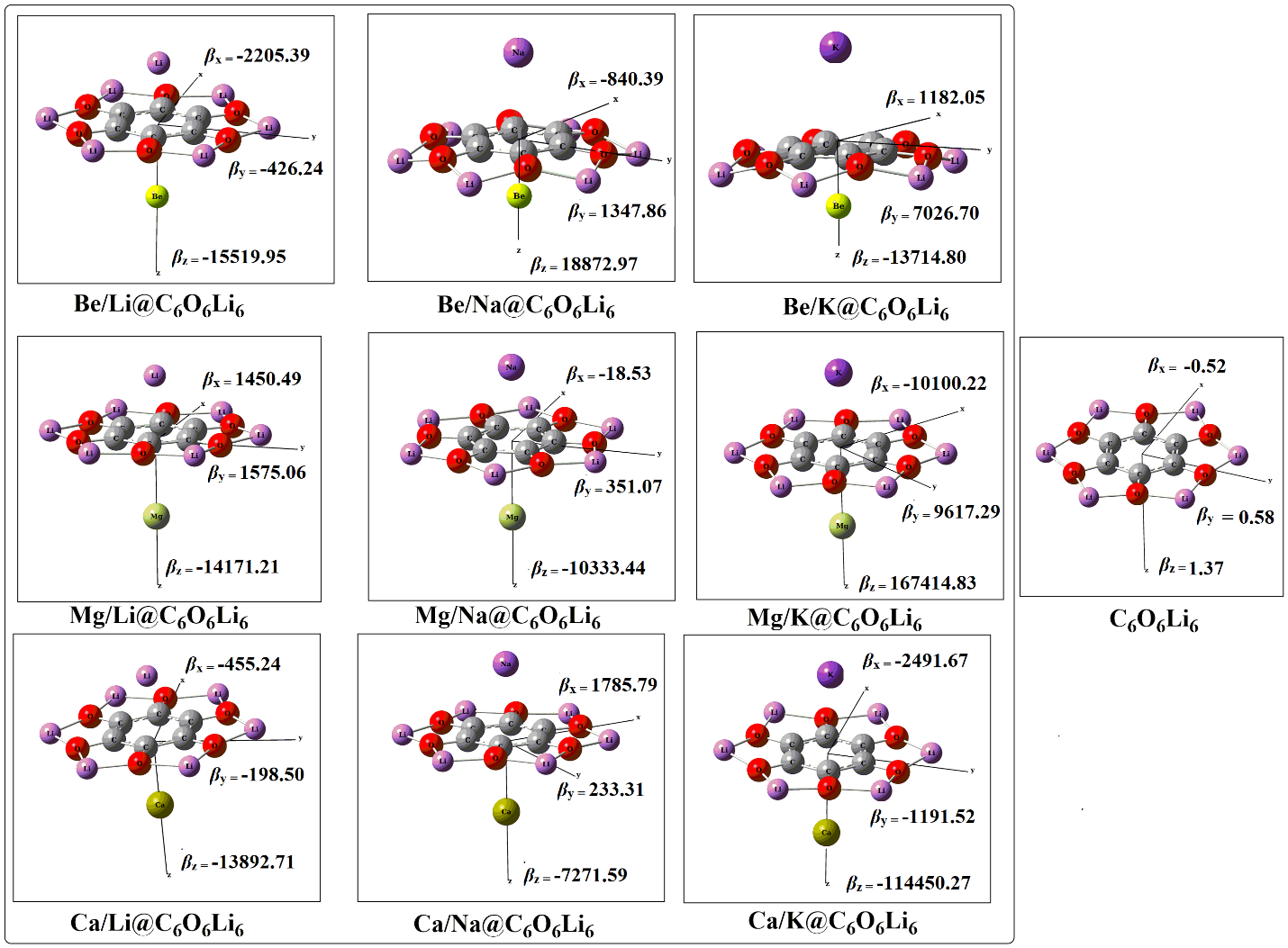


**SI. Figure 1:** Graphical representation of optimized structuresfor pure C_6_O_6_Li_6_ and dual alkali/alkaline earth metals doped C_6_O_6_Li_6_ electrides having hyperpolarizaibility values along x, y and z axsis.





**SI. Figure 2:** Graphical representation of LUMOs of pure C_6_O_6_Li_6_ and dual alkali/alkaline earth metals doped C_6_O_6_Li_6_ electrides (isovalue = 0.05).





**SI. Figure 3:** Graphical representation of Natural bond orbitals (NBOs) of pure C_6_O_6_Li_6_ and dual alkali/alkaline earth metals doped C_6_O_6_Li_6_ electrides (isovalue = 0.05).































**SI. Figure 4:** Graphical representation of Natural transition orbitals (NTOs) of pure C_6_O_6_Li_6_ and dual alkali/alkaline earth metals doped C_6_O_6_Li_6_ electrides (isovalue = 0.05).

***1.1 Two level model of dual alkali and alkaline earth metals doped C_6_O_6_Li_6_ electrides***

To further calculate the internal parameters which influence the *β*_o_ values of excess electron electrides systems, we evaluated *β*_z_ values by implementing two level model with the use of MultiWfn software [69]. Following is the mathematical form of two-level model.

𝛽_o_ ≈ ∆µ × 𝑓_o_/∆E^3^ … …… … … … … … … … … … … … … ………………..12

Above equation reveals that crucial excitation energies are inversely related to *β*_o_ but it has linear increment with variation in dipole moment and oscillation strength. Two level model reveals that the variation of excitation energy has the most prominent role in evaluating the hyperpolarizabilities of designed complexes [70]. The excitation energies of Be/Li@C_6_O_6_Li_6_, Be/Na@C_6_O_6_Li_6_, Be/K@C_6_O_6_Li_6_, are 1.57 eV, 1.79 eV and 1.46 eV, respectively. In Mg/Li@C_6_O_6_Li_6_, Mg/Na@C_6_O_6_Li_6_ and Mg/K@C_6_O_6_Li_6_, the excitation energies are 1.56 eV, 1.70 eV, 1.44 eV, respectively. In Ca/Li@C_6_O_6_Li_6_, Ca/Na@C_6_O_6_Li_6_ and Ca/K@C_6_O_6_Li_6_, the excitation energies are 1.68 eV, 1.78 eV and 1.14 eV, respectively. The increasing trend in crucial excitation energies of doped electrides is comparable to that of the first hyperpolarizability values in each series of alkaline earth metals (Be, Mg, Ca) doped C_6_O_6_Li_6_ (B = Li, Na, K). *β*_o_ of Mg/K@ C_6_O_6_Li_6_ is 1.68×10^5^ au which has excitation energy of 1.44 eV. Here, we observed the same behavior if the excitation energy is smaller for an electrides then *β*_o_ for this complex is also high.

We see that beside excitation energy, other factors which influence the values of hyperpolarizability are oscillation strength and dipole moment of that particular complex. Table 3 show that trend in the increasing values of oscillation strength and dipole moment are opposite to that of hyperpolarizability values for all A/B@C_6_O_6_Li_6_ (A = Be, Mg, Ca, B = Li, Na, K) electrides. Thus, the major factor which contribute to the hyperpolarizability is the excitation energies. We also calculated *β*_z_ values and observed similar variational trend of *β*_z_ values as we seen in *β*_o_ values for dual alkali/alkaline earth metals doped C_6_O_6_Li_6_ electrides. Sunaina *et al*. observed similar comparable trend of *β*_o_ and *β*_z_ values for alkali metals doped C_6_O_6_Li_6_ organometallic [52].

**SI. Table 3:** Oscillator strength (ƒₒ), transition energy (∆E in eV), variational dipole moment Z component between ground and crucial excited state (∆*µ* in Debye), and wavelength (λ_max_ in nm) of pure C_6_O_6_Li_6_ and Mg/B@C_6_O_6_Li_6_ (B = Li, Na, K) complexes. interaction energies (E_int_ in kcal mol^-1^) and vertical ionization energies (IE_v_ in eV) and

| **Parameters** | ***ƒ*_o_** | **∆E** | **∆*µ*** | **λ_max_** |
| --- | --- | --- | --- | --- |
| **C_6_O_6_Li_6_** | 0.03 | 2.42 | 0.01 | 515 |
| **Be/Li@C_6_O_6_Li_6_** | 0.13 | 1.57 | 5.71 | 718 |
| **Be/Na@C_6_O_6_Li_6_** | 0.17 | 1.79 | -3.05 | 841 |
| **Be/K@C_6_O_6_Li_6_** | 0.19 | 1.46 | -4.67 | 792 |
| **Mg/Li@C_6_O_6_Li_6_** | 0.14 | 1.56 | 5.45 | 783 |
| **Mg/Na@C_6_O_6_Li_6_** | 0.17 | 1.7 | 5.44 | 729 |
| **Mg/K@C_6_O_6_Li_6_** | 0.14 | 1.44 | -4.7 | 1622 |
| **Ca/Li@C_6_O_6_Li_6_** | 0.175 | 1.68 | 5.72 | 743 |
| **Ca/Na@C_6_O_6_Li_6_** | 0.17 | 1.78 | 4.15 | 701 |
| **Ca/K@C_6_O_6_Li_6_** | 0.092 | 1.14 | 5.16 | 828 |
